# MAKING IT WORK: SECURING A GERONTOLOGY INTERNSHIP FOR THE NONTRADITIONAL DISTANCE EDUCATION LEARNER

**DOI:** 10.1093/geroni/igz038.1973

**Published:** 2019-11-08

**Authors:** Katherine Im

**Affiliations:** UMUC, Adelphi, Maryland, United States

## Abstract

Some gerontology programs have required students to complete internships in order to satisfy the “interactional” Category II Competencies for gerontology as outlined by AGHE in 2014. For many non-traditional learners, a required internship in gerontology poses major challenges that may contribute to declines in enrollment and retention. Non-traditional learners are often older, employed, and have multiple responsibilities that compete for their time. This is especially true for students who attend university from a distance. This presentation reports the challenges faced by non-traditional, online students at a large state university who have an interest in gerontology. For these learners, the internship requirement is a deterrent to becoming a gerontology major. Challenges include student constraints, identifying potential placement sites in remote locations, and negotiating a complex administrative process. I review the programmatic measures taken to address these challenges and discuss the ways in which the internship process can be facilitated for non-traditional students. By developing a better process for securing internships, students have more freedom to focus on workplace learning. In addition, prospective gerontology students may come to regard the internship as an excellent opportunity for professional growth rather than a burden , thereby improving retention and interest in the major.

